# Genomic variation in tomato, from wild ancestors to contemporary breeding accessions

**DOI:** 10.1186/s12864-015-1444-1

**Published:** 2015-04-01

**Authors:** José Blanca, Javier Montero-Pau, Christopher Sauvage, Guillaume Bauchet, Eudald Illa, María José Díez, David Francis, Mathilde Causse, Esther van der Knaap, Joaquín Cañizares

**Affiliations:** Institute for the Conservation and Improvement of Agricultural Biodiversity (COMAV), Polytechnic University of Valencia, Camino de Vera 8E, 46022 Valencia, Spain; INRA, UR 1052 Unité de Génétique et Amélioration des Fruits et Légumes, Domaine Saint-Maurice, 67 Allée des Chênes CS60094, 84143 Montfavet Cedex, France; Syngenta seeds, 12 chemin de l’hobit, 31790 Saint-Sauveur, France; Department of Horticulture and Crop Science, The Ohio State University/Ohio Agricultural Research and Development Center, Wooster, OH 44691 USA

**Keywords:** *Solanum lycopersicum*, *Solanum pimpinellifolium*, SolCAP array, Origin, Variability, Genome, Fruit size genes, Domestication

## Abstract

**Background:**

Domestication modifies the genomic variation of species. Quantifying this variation provides insights into the domestication process, facilitates the management of resources used by breeders and germplasm centers, and enables the design of experiments to associate traits with genes. We described and analyzed the genetic diversity of 1,008 tomato accessions including *Solanum lycopersicum* var. *lycopersicum* (SLL), *S. lycopersicum* var. *cerasiforme* (SLC), and *S. pimpinellifolium* (SP) that were genotyped using 7,720 SNPs. Additionally, we explored the allelic frequency of six loci affecting fruit weight and shape to infer patterns of selection.

**Results:**

Our results revealed a pattern of variation that strongly supported a two-step domestication process, occasional hybridization in the wild, and differentiation through human selection. These interpretations were consistent with the observed allele frequencies for the six loci affecting fruit weight and shape. Fruit weight was strongly selected in SLC in the Andean region of Ecuador and Northern Peru prior to the domestication of tomato in Mesoamerica. Alleles affecting fruit shape were differentially selected among SLL genetic subgroups. Our results also clarified the biological status of SLC. True SLC was phylogenetically positioned between SP and SLL and its fruit morphology was diverse. SLC and “cherry tomato” are not synonymous terms. The morphologically-based term “cherry tomato” included some SLC, contemporary varieties, as well as many admixtures between SP and SLL. Contemporary SLL showed a moderate increase in nucleotide diversity, when compared with vintage groups.

**Conclusions:**

This study presents a broad and detailed representation of the genomic variation in tomato. Tomato domestication seems to have followed a two step-process; a first domestication in South America and a second step in Mesoamerica. The distribution of fruit weight and shape alleles supports that domestication of SLC occurred in the Andean region. Our results also clarify the biological status of SLC as true phylogenetic group within tomato. We detect Ecuadorian and Peruvian accessions that may represent a pool of unexplored variation that could be of interest for crop improvement.

**Electronic supplementary material:**

The online version of this article (doi:10.1186/s12864-015-1444-1) contains supplementary material, which is available to authorized users.

## Background

The domestication process of crop plants led to dramatic phenotypic changes in many traits that result from changes in the genetic makeup of the wild species ancestors [1,2]. The analyses of genomic variation and the structure of genetic diversity of cultivated crops and their wild relatives provides insights into the history of domestication, adaptation to local environments, and breeding [3,4]. The resulting analyses offer valuable information for germplasm management and the exploitation of natural variation to improve crops.

Cultivated tomato (*Solanum lycopersicum* L.) (SL) is a member of the family *Solanaceae*, genus *Solanum* L., section *Lycopersicon* [5]. Its wild relatives are native to western South America, including the Galapagos Islands. *S. pimpinellifolium* L. (SP) is thought to be the closest wild ancestor to cultivated tomato [5-7]. SP accessions are found in Coastal Peru and Ecuador and are divided in three main genetic groups corresponding to the environmental differences found in the coastal regions of Northern Ecuador, in the montane region of Southern Ecuador and Northern Peru, and the coastal region of Peru [8,9].

*S. lycopersicum* is divided into two botanical varieties: *S. l.* var. *cerasiforme* (Dunal) Spooner, G.J. Anderson & R.K. Jansen (SLC) and *S. l.* var. *lycopersicum* (SLL). SLC is native to the Andean region encompassing Ecuador and Peru, but it is also found in the subtropical areas all over the world [10]. SLC grows either as a true wild species, in home gardens, along roads, sympatrically with tomato landraces, or as a cultivated crop [9]. SLC thrives in the humid environments of Ecuador and Peru at the eastern edge of the Amazon basin whereas SP occupies the drier Peruvian coasts and valleys and the wetter Ecuadorian coast [9,11,12]. Although there is no reproductive barrier between SP and SLC [13], the Andes mountains impose strong physical and ecological barriers for cross reproduction among these species.

Many details of tomato domestication remain debated, especially regarding the role of SLC in this process. The South American SLC native to the Ecuadorian and Peruvian Andes has been proposed to be an evolutionary intermediate between SP and cultivated SLL [6,9,14] or, alternatively, an admixture resulting from the extensive hybridization between SP and SLL [15,16]. The location of tomato domestication also remains uncertain. Both Mesoamerica [14] or Ecuador and Northern Peru, near the center of origin of SP [17], have been proposed as the center of domestication. If the former were true, SLC would have had to migrate north to Mesoamerica as a wild or weedy species, where it would have been domesticated into SLL. Instead, a two-step domestication process has been proposed for tomato [9]. The first step would have consisted of a selection from SP or primitive SLC by early farmers resulting in the Ecuadorian and Northern Peruvian SLC. The second step likely occurred in Mesoamerica, and consisted of further selection from these pre-domesticated SLC after their migration from Ecuador and Peru. This second step completed the domestication process of tomato. Genetic data confirmed that European SLL accessions originated from Mesoamerica and constitute the genetic base of the SLL vintage varieties [9]. It has also been proposed that a genetic bottleneck was associated with the migration of SLL from Mesoamerica to Europe [18-20]. Blanca et al. [9] proposed that the main bottleneck happened during the migration from Peru and Ecuador.

Extensive breeding efforts have modified tomato over the last 100 years. Breeding goals were focused on improving SLL for disease resistance, adaptation to diverse production areas, yield and uniformity. These efforts resulted in the introduction of many introgressions from SP and more distant tomato relatives [21], leading to a broadening of the genetic diversity of SLL [21-23]. Another consequence of these breeding programs was the selection for specific traits that are characteristic of the fresh and processing markets which has led to further diversification and genetic differentiation among market classes.

The traits that most likely have been selected during the domestication of tomato were fruit weight and, to a lesser extent, shape. In recent years, several genes affecting these traits have been identified [24-29]. As the underlying polymorphism causing the change in allele function for all these genes is known, the presence of the derived and ancestral alleles is easily sampled. For example, in vintage SLL the majority of the shape diversity is explained by the derived alleles of the *FAS*, *SUN*, *OVATE* and *LC* genes [30]. What is not well understood is when and where these alleles arose and how they spread through the germplasm. Quantifying the allele frequency of the loci among the SP and SLC populations will help to elucidate the process of selection that is at the foundation of tomato domestication.

The aim of this study was to better delineate the evolutionary history of tomato including its domestication. By using a dataset with over 7,000 SNPs and 1,008 accessions of SP, SLC and SLL we aim to compare and contrast the genome-wide molecular diversity of populations spanning the entire red-fruited clade. Additionally, the allele frequency of six fruit weight and shape genes have been measured in order to elucidate the domestication process.

## Methods

### Plant material and passport data

We analyzed 1,008 tomato accessions from the species representing the red-fruited clade of tomato (Additional file 1: Table S1). Of these, 912 corresponded to accessions genotyped in studies conducted at COMAV, Spain [9], through the Solanaceae Coordinated Agricultural Project (SolCAP) in the USA [31] and INRA, France [32]. These data sets were combined with an additional set of 96 accessions originating from vintage and processing germplasm genotyped in Ohio (62), and from the COMAV collection (34). Altogether, these 1,008 accessions represent 952 uniquely named accessions. Several accessions were independently genotyped in different experiments. For example, Moneymaker was represented several times and these duplicates were used for quality control of the genotyping results between the laboratories. The number of uniquely named accessions per species, according to their passport data, were: *Solanum lycopersicum* var. *lycopersicum* (SLL; 530 accessions), *S. l.* var. *cerasiforme* (SLC; 316 accessions), *S. pimpinellifolium* (SP; 145 accessions), *Solanum galapagense* S.C.Darwin & Peralta (SG; 4 accessions), *Solanum neorickii* D.M.Spooner, G.J.Anderson & R.K.Jansen (SN; 1 accession), *Solanum chmielewski* (C.M.Rick, Kesicki, Fobes & M.Holle) D.M.Spooner, G.J.Anderson & R.K.Jansen (SChm; 1 accession), crosses between *S. lycopersicum* and *S. pimpinellifolium* (SL x SP; 10 accessions), and one hybrid between *S. l. lycopersicum* and *S. pennellii*. The hybrids were included to determine the ability of detecting heterozygous SNPs with the genotyping platform.

A unified passport classification, which includes species name, collection site and use, was compiled for all accessions based on the information retrieved from the different sources and donors (Additional file 1: Table S1). For SP and SLC, the passport classification mainly reflected the collection site. An additional category for SLC was introduced as “SLC commercial cherry” to group the SLC accessions with a commercial purpose. For SLL, the vintage, landrace and heirloom categories were grouped together and classified collectively as vintage consistent with the nomenclature of Williams and St. Clair [19]. Additionally, a category was created in SLL to include the early breeding lines such as Moneymaker and Ailsa Craig. The SLL accessions derived from crop improvement programs currently active (i.e. contemporary to the time of writing) were categorized based on use (fresh market or processing) and location of breeding. Overall, sufficient information was available for 84% of the accessions to classify them beyond the species level. In cases where this was not possible, the passport classification only reflected the species (i.e., SP, SLC or SLL). For 48.3% of the accessions, geographic location information was available in the form of Global Positioning System (GPS) coordinates or from the location of its collection site (Additional file 1: Table S1).

### Genotyping and data set merging

All samples were genotyped using the Tomato Infinium Array (Illumina Inc., San Diego, CA, USA) developed by the United States Department of Agriculture (USDA) funded SolCAP project (http://solcap.msu.edu/). The SolCAP SNP discovery work-flow was described [33], as were details of the array [23]. The genotyping array contained probes for 8,784 biallelic SNPs. These SNPs represented a highly filtered and selected set, based on transcriptome sequence for SLL, SLC, and SP, optimized for polymorphism detection and distributed throughout the genome. Of these, 7,720 SNPs (88%) passed manufacturing quality control [23]. All SNPs on the array have been incorporated into the Solanaceae Genome Network database (http://solgenomics.net/), the SNP annotation file is available (http://solcap.msu.edu/tomato_genotype_data.shtml), and sequences are available through the Sequence Read Archive (SRA) at the National Center for Biotechnology Information (study summary SRP007969; accession numbers SRX111556, SRX111557, SRX111558, SRX111845, SRX111848, SRX111849, SRX111850, SRX111853, SRX111857, SRX111858, SRX111859, SRX111862, SRX111861).

Genomic DNA was isolated from fresh young leaf tissue. DNA concentrations were quantified using the PicoGreen assay (Life Technologies Corp., Grand Island, NY, USA) and diluted to 50 ng/μl in TE buffer (10 mM Tris–HCl pH 8.0, 1 mM EDTA). Genotyping was performed using 250 ng of DNA per accession following the manufacturer’s recommendations. The intensity data were analyzed in GenomeStudio version 1.7.4 (Illumina Inc., San Diego, CA, USA). The automated cluster algorithm generated from the SolCAP project was used to obtain initial SNP calls. Visual inspection was used to assess the default clustering of each SNP, and calls were modified when the default clustering of a SNP was not clearly defined.

There are three methods for SNP calling for the Illumina Infinium array: relative to the reference (also known as customer), the design (also known as Illumina) or the TOP strand (a designation based on the polymorphism itself and its flanking sequence). To merge data sets from three different laboratories that had used different SNP calling methods, we developed a Python script to facilitate detection, reorientation and merging of the data such that all SNPs are called relative to the design strand (the script is available upon request to J. Blanca).

### Selection of SNPs for downstream analyses

The accessions were genotyped with 7,720 SNPs (Additional file 2: Table S2) that passed the manufacturing quality control and constituted the raw data set. Of those, we removed 240 markers (3.1%) that had more than 10% missing data and 1137 (14.7%), which had a major allele frequency above 0.95. For all analyses, except for the rarefaction and the linkage disequilibrium (LD), SNPs that mapped closer than 0.1 cM were removed as well, yielding a final dataset of 2,313 markers uniformly distributed across the genome. This filtering was done in order to avoid an overestimation of polymorphism and genetic distances among populations due to genomic introgressions from wild relatives. For this purpose a minimum genetic distance of 0.1 cM was chosen as a trade-off between the number of markers left for the analysis and the LD minimization. Genetic distances were based on the genetic maps of Sim et al. [23].

### Genetic classification and sample filtering

Principal Component Analyses (PCA) were used to explore the patterns of genomic variation in the entire collection without considering the *a priori* classification based on passport data (i.e., species, location and use). A three level classification scheme, based on a series of hierarchical PCAs, was used to define genetic groups within species and genetic subgroups within genetic groups. PCAs were performed with the smartPCA application included in the Eigensoft 3.0 package [34,35]. This genetic classification was used in the subsequent analyses unless mentioned otherwise.

Pairwise genetic distances were computed among accessions within each group at each level of the hierarchical classification. Kosman and Leonard’s distance method [36] was used and a violin plot was produced for each hierarchy level using the R package ‘vioplot’ [37].

When an accession was genotyped more than once and both genotypes were inconsistent (e.g., both samples were classified in different subgroups in the PCA) all data for the accession was removed from the analysis (see Additional file 1: Table S1), unless it was clear based on the passport information, which genotype was correct (e.g., two entries from the same SLC accession collected in Peru, one grouping with other Peruvian accessions and another grouping with the mixture group). In total 8 genotypes out of the 1,008 were removed due to inconsistent data. We assume that these rare inconsistencies were related to uncontrolled cross pollinations or seed mixing during regeneration.

Genetic distances among samples of the same uniquely named accession were evaluated (see above) to check the reproducibility between genotyping datasets coming from different laboratories. For the genetic analyses, unless stated to the contrary, only one randomly chosen genotype representative of the uniquely named accessions was used.

### Diversity and genetic differentiation

For polymorphic loci with a major allele frequency lower than 0.95 (P95), the expected (H_e_) and observed (H_o_) heterozygosity were calculated using custom scripts for each hierarchy of the genetic classification. Differentiation among genetic subgroups was explored by calculating differentiation index D_est_ [38] using custom scripts and F_st_ using Arlequin v. 3.5.1.3 [39]. Only groups with at least 5 individuals were considered for genetic diversity estimates and mixture groups (SP mixture, SLC mixture and mixture) were not included in these analyses. Statistical significance of D_est_ and F_st_ was assessed after 1,000 permutations.

An unrooted network was built based on the genetic differentiation matrix using the Neighbor-net algorithm implemented in SplitsTree v.4.13.1 [40]. Additionally, a neighbor-joining tree was created using the same distance matrix. Bootstrap values were obtained from 1,000 trees. The tree was built using functions included in PyCogent v. 1.5.3 library [41].

Allelic richness and private allelic richness (private alleles are defined as alleles found exclusively in a single population) were estimated using the rarefaction method implemented in the software ADZE [42]. LD was calculated using TASSEL v.4.0 [43]. Pairwise *r*^2^ was obtained for all markers within each chromosome and data was fitted using a local polynomial regression fitting (LOESS) [44] implemented in R v. 3.0.1 [45]. Rarefaction and LD analyses were performed using genetic groups defined by PCA and network analysis. These groups are defined as follows: SP, SLC Ecuador and Northern Peru, SLC non Andean, SLL vintage and SLL contemporary (split for some analyses into SLL processing and SLL fresh).

### Isolation by distance

Correlations between genetic, geographic and climatic distances were analyzed to infer patterns of isolation by distance or the effect of ecological conditions on the genetic structure. Pairwise genetic distances between accessions were computed using Kosman and Leonard’s distance method [36]. Pairwise geographic distances were calculated when GPS information was available using the haversine formula [46]. Climatic data for accessions with GPS coordinates was obtained using the R package ‘raster’ [47]. Current climatic data interpolated from 1950 to 2000 was obtained from worldclim (http://www.worldclim.org) at 30 arc-seconds resolution (approx. 1 km). A PCA was carried out with all the climatic information and the resulting scores were used to obtain the pairwise climatic distances based on a Euclidean metric. Significance of the correlations between distance matrices was assessed with a Mantel test based on 1,000 permutations implemented by the PyCogent Python library [41]. A density plot for each distance comparison was created using the *kde2d* function in the R ‘MASS’ package [45].

### Phylogenetic analysis

A phylogenetic tree was built with SNAPP [48] to infer the evolutionary history of the tomato species in the Andean region encompassing Ecuador and Peru. SNAPP, which is part of the BEAST package [49], is a recently developed method that allows reconstructing the species tree from unlinked SNPs by using a finite-sites model likelihood algorithm within a Bayesian Markov chain Monte Carlo (MCMC). A MCMC chain was run for 2,000,000 steps with a sampling interval of 1,000 and a burn-in of 25%. Convergence of posterior and likelihood distributions, and number of estimated sample size for model parameters were assessed using Tracer v.1.5 [50]. Due to the high computational demands of SNAPP, only one accession per genetic subgroup was used. For the same reason, not all genetic subgroups were considered; only SP and Peruvian, Ecuadorian and Mesoamerican SLC accessions were included. Three outgroup species were also included, namely *S. galapagense, S. neorickii* and *S. chmielewski*.

### Fruit weight and shape genes genotyping

Six markers that distinguish wild type and causal derived alleles of the fruit shape loci *(sun*, *ovate*, *fas* and *lc*) as well as the fruit weight loci (*fw2.2* and *fw3.2*) were genotyped (Table 1 and Additional file 1: Table S1). *lc* (locule number) and *fas* (fasciated) control the number of locules, an important feature affecting fruit weight as well as shape.The gene *lc* is hypothesized to be an ortholog of WUSCHEL which is required to maintain stem cell identity [28]. The *fas* mutation affects a YABBY2 transcription factor which encodes a member of the family regulating organ polarity [27,51]. Two genes exhibit a major effect on fruit shape namely *sun* [26] and *ovate* [25], positive and a negative regulators of growth, respectively. The fruit weight gene *fw2.2* negatively controls cell division and encodes a member of the Cell Number Regulator (CNR) family [24,52]. *fw3.2* encodes an ortholog of KLUH, a P450 enzyme which increases weight through increased cell number in pericarp and septum tissues [29].

**Table 1:** **Fruit shape and size marker information**

**Gene**	**Primer sequence (5′ to 3′)**	**Polymorphism**	**Restriction enzyme**	**Wild-type allele size (bp)**	**Cultivated allele size (bp)**	**Reference**
*FW2.2/CNR*	CATATAAAGTGTACTGACCGTCA	SNP	*Tsp*45I	168	149	This paper
	CTGTCCTATTCAAGAGGTAAATGAG					
*FW3.2/SlKLUH*	AAAGTCGAATAAATTAGATGAACTTGA	SNP	*Hpy*188I	326	304	Chakrabarti et al. [29]
	ATTGGGTCTCTCCTCGCTCT					
*LC*	GCCGAACACATCAACATTTC	SNP	*Hin*dIII	260	235	*Muños et al. [28]
	CCTTTTCCTAAAAGATTTGGCATGAAG					
*FAS*	CCAATGATAATTAAGATATTGTGACG	Inversion	-	466	335	Rodríguez et al. [30]
	ATGGTGGGGTTTTCTGTTCA					
	CAGAAATCAGAGTCCAATTCCA					
*OVATE*	AAGCTGATACCGTGTAGTGTGG	SNP	*Dde*I	122	109	*Rodriguez et al. [30]
	AATGCTTTCCGTTCAACGAC					
*SUN*	TTTACCCGATGTGAAAACGA	RFLP	*Eco*RV		An additional 4.3-kb fragment	Xiao et al. [26]
	CATCAATAGTCCAAGGGGAAA					

*Marker that is modified from the original.

All markers, except *sun,* were genotyped by amplification using standard PCR following previously published methods [30]. PCR products were scored directly (*fas*) or after restriction enzyme-digestion (*lc*, *ovate*, *fw2.2*, *fw3.2*) by electrophoresis on 3% TBE (110 mM Tris, 90 mM boric acid, 2.5 mM EDTA) agarose gels. The *sun* duplication was scored as an RFLP using standard Southern blotting and hybridization protocols [53].

## Results

### Genetic structure of the tomato accessions

**Figure 1:**
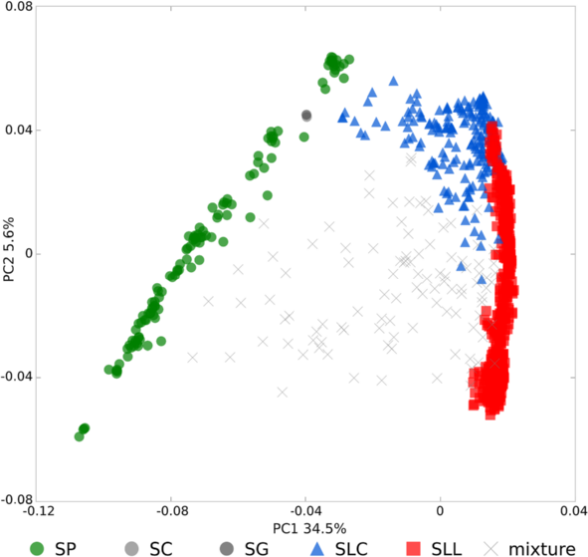
**Principal component analysis using all 952 uniquely named accessions and based on 2,313 markers.**

To detect patterns of genetic structure within the collection, we conducted a global PCA (Figure 1) using 2,313 selected SNPs. The graphical pattern of the first two principal components (PCs) is suggestive of an arch structure with the three edges corresponding to SP, SLC and SLL respectively. The small-fruited wild relative SP forms the left side, differentiated along both PCs. SLC corresponded to the top of the arch and was also distributed along both PCs albeit less clearly than SP. SLL accessions are differentiated only along PC2, forming the right edge (positive PC1, distributed PC2). Additionally, a group of genotypes appeared in between the three main groups and they have been classified as mixture. The accessions in this region include all ten artificial SLL x wild species hybrids and the accessions BGV007985, BGV012625 and LA1909 are already classified as interspecific hybrids in their passport data, thus we have called this group “mixture”. The SP category was the most genetically diverse group (H_e_ = 0.21), followed by SLC (H_e_ = 0.17) and SLL (H_e_ = 0.12) (Table 2).

**Table 2:** **Summary of genetic-based classification: observed and expected heterozygosity (H**_**o**_ **, H**_**e**_ **), percentage of markers with a major frequency allele lower that 0.95 (P95) and number of individuals (N) for the species, groups and subgroups of the genetic-based classification (subgroups with less than 5 accessions are not listed)**

**Species**	**Group**	**Subgroup**	**H**_**0**_	**H**_**e**_	**P95**	**N**
SP			0.042	0.205	0.557	112
	SP Ecuador		0.019	0.085	0.269	17
		SP Ecuador 1	0.009	0.059	0.169	10
		SP Ecuador 2	0.037	0.084	0.207	5
	SP Montane		0.027	0.132	0.353	12
		SP Montane 1	0.007	0.060	0.133	5
		SP Montane 2	0.041	0.139	0.364	7
	SP Peru		0.050	0.151	0.444	83
		SP Peru 1	0.095	0.166	0.437	13
		SP Peru 2	0.059	0.138	0.405	18
		SP Peru 3	0.086	0.116	0.359	9
		SP Peru 4	0.035	0.089	0.251	14
		SP Peru 7	0.046	0.093	0.243	6
		SP Peru 8	0.018	0.072	0.192	6
		SP Peru 9	0.011	0.040	0.098	7
SLC			0.023	0.170	0.551	221
	SLC Ecuador		0.038	0.188	0.522	45
		SLC Ecuador 1	0.024	0.125	0.357	12
		SLC Ecuador 2	0.035	0.174	0.492	17
		SLC Ecuador 3	0.004	0.095	0.241	6
		SLC Vintage	0.087	0.168	0.486	10
	SLC Peru		0.023	0.177	0.541	43
		SLC Peru 1	0.017	0.122	0.324	8
		SLC Peru 2	0.019	0.142	0.492	20
		SLC Peru 3	0.031	0.199	0.620	15
	SLC SP Peru	SLC SP Peru	0.031	0.116	0.323	7
	SLC non_Andean		0.012	0.110	0.317	119
		SLC Colombia	0.028	0.101	0.293	7
		SLC Costa Rica	0.024	0.090	0.257	8
		SLC Mesoamerica	0.009	0.079	0.262	37
		SLC Asia	0.007	0.071	0.197	14
		SLC other	0.010	0.095	0.237	49
	SLC 1	SLC 1	0.087	0.164	0.512	7
SLL			0.012	0.124	0.346	492
	SLL vintage		0.010	0.094	0.257	172
		SLL Mesoamerica	0.021	0.102	0.279	33
		SLL vintage 1	0.007	0.082	0.223	120
		SLL early breed	0.006	0.064	0.229	14
		SLL vintage 2	0.008	0.097	0.231	5
	Contemporary SLL		0.012	0.115	0.310	306
	SLL fresh		0.010	0.091	0.272	128
		SLL vintage/fresh	0.013	0.087	0.253	54
		SLL fresh 1	0.006	0.069	0.208	69
		SLL fresh 2	0.043	0.063	0.148	5
	SLL processing 1		0.013	0.096	0.265	165
		SLL processing 1 1	0.011	0.094	0.264	37
		SLL processing 1 2	0.012	0.084	0.003	124
	SLL processing 2	SLL processing 2	0.012	0.056	0.132	13

**Figure 2:**
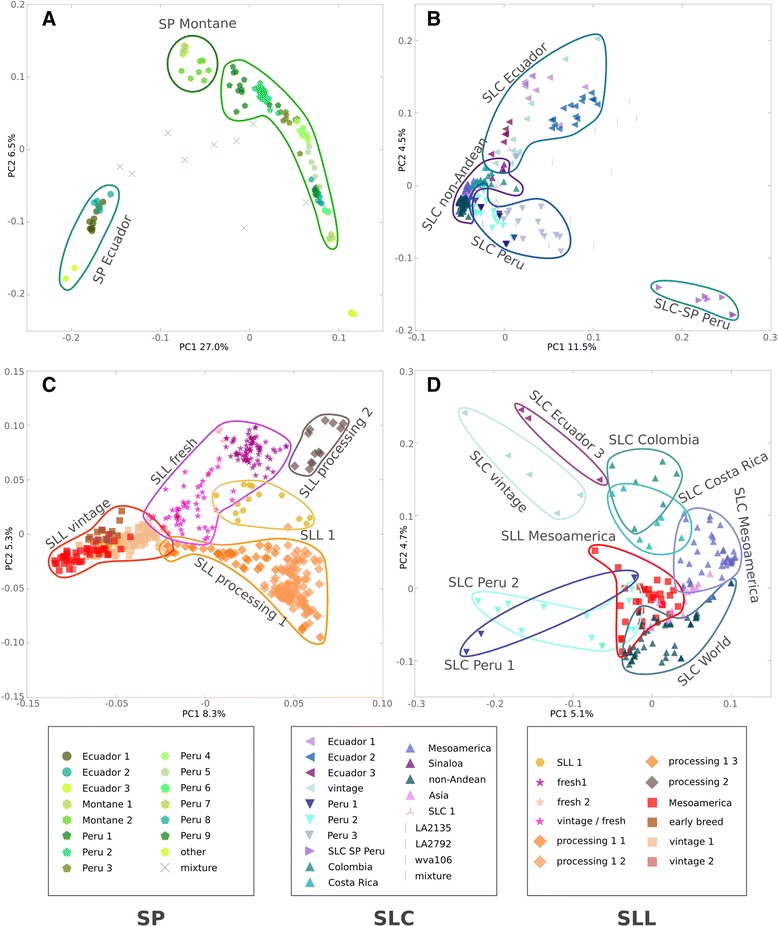
**Principal Component Analysis for SP, SLC, SLL and SLL Mesoamerica and genetically close SLC subgroups.** **(A)** SP, **(B)** SLC, **(C)** SLL and **(D)** SLL Mesoamerica and genetically close SLC subgroups. Solid lines encircle the main genetic groups and different colors and markers represent genetic subgroups.

To identify clusters within each species (i.e., genetic groups) and sub-clusters within each cluster (i.e., genetic subgroups), additional PCAs were conducted in a hierarchical fashion with the accessions belonging to the same species (Figure 2 and Additional file 3: Figure S1, Additional file 4: Figure S2, Additional file 5: Figure S3, Additional file 1: Table S1). For SP, the first two PCs (explaining 33.5% of the total variance) showed that the SP Ecuador, that comprises Northern Ecuadorian accessions, formed a separate genetic group from the other SP accessions (Figure 2A and Additional file 3: Figure S1). These Ecuadorian accessions were further subdivided into three genetic subgroups: Ecuador 1, Ecuador 2 and Ecuador 3 (Additional file 3: Figure S1A and B). The remaining SP accessions were divided into two genetic groups: Peru (corresponding mainly to Coastal Peru and Northern Montane Peru) and Montane (Southern Ecuadorian Montane accessions) (Figure 2A and Additional file 3: Figure S1). Montane accessions were further subdivided into two genetic subgroups (Montane 1 and Montane 2), whereas the Peruvian accessions clustered into 9 categories (Additional file 3: Figure S1C- F). Accessions located in an intermediate position in the PCA were classified as SP mixture, and likely represent admixtures between SP accessions from different groups (Figure 2A). These admixtures could be from naturally occurring hybridizations or the result of accidental outcrossing events during the handling of the accessions in germplasm collections or regeneration in seed banks. The genetic diversity among the three SP groups ranged from H_e_ = 0.09 (Ecuadorian SP) to H_e_ = 0.15 (Peruvian SP) (Table 2).

For SLC, the first two PCs explained 16.0% of the total variance and showed a clustering based on geography (Figure 2B; Additional file 3: Figure S1). The Ecuadorian and Peruvian SLC formed two non-overlapping clusters in the PCA representation and showed a higher genetic diversity compared to SP Ecuador and SP Montane (SLC Ecuador H_e_ = 0.19 and SLC Peru H_e_ = 0.18, Table 2). An SLC group which included accessions from all over the subtropical regions of the world was called SLC non-Andean, and was located between the two Andean clusters (Figure 2B). A distinct cluster named SLC-SP Peru was identified and composed of accessions from Southern Peru.

**Figure 3:**
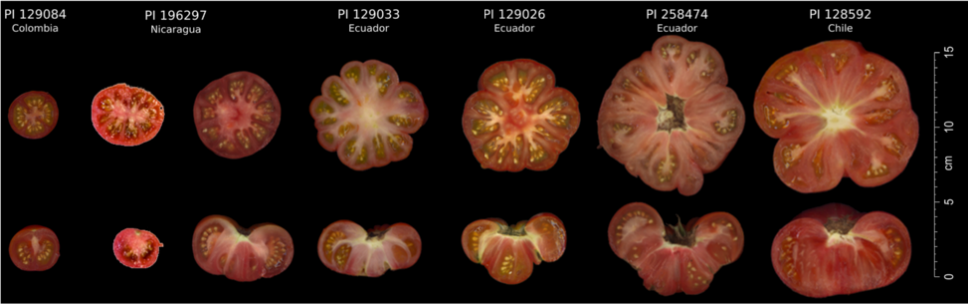
**Tomatoes from SLC vintage subgroup (source** **http://www.ars.usda.gov** **).**

Each SLC genetic group could be further subdivided based on genetic structure. Ecuadorian SLC was split into four subgroups, three that divided Ecuador latitudinally (Additional file 4: Figure S2A and B, Additional file 6: Figure S4) and one that was named SLC vintage since it mainly included accessions collected from South American markets as vintage tomatoes. Interestingly, the SLC vintage accessions often featured big fruits with many locules, a trait that may have been selected early for cultivation and consumption (Figure 3). The SLC vintage accessions clustered closely, but separately, relative to the three Ecuadorian genetic subgroups (Additional file 4: Figure S2A and B). The Peruvian SLC was divided into three subgroups that were named from north to south as Peru 1, Peru 2, and Peru 3. The SLC non-Andean group was subdivided into: Colombia, Costa Rica, Mesoamerica, Sinaloa (Mexico), South East Asia and Other representing the rest of the subtropical regions of the world (mainly Europe, Africa and South American nations outside of Colombia, Ecuador and Peru).

Similarly to SP, SLC accessions without a clear genetic clustering and without a common geographic origin were classified as SLC mixture. These mixture accessions were distributed between the Peruvian and Ecuadorian SLC clusters in the PCA (Figure 2B). In addition, closely related to the SLC non-Andean were seven accessions with no obvious relationship according to the passport data and were referred to as SLC 1.

The PCA for the SLL accessions showed that the first two PCs (13.6% of the total variance) separated five main genetic groups: vintage, fresh, processing 1, processing 2 and SLL 1 (Figure 2C). All SLL groups had low diversity (H_e_ = 0.06-0.10) compared with the Peruvian and Ecuadorian SLC (H_e_ = 0.188-0.177) (Table 2). The SLL vintage group was divided into subgroups that were differentiated using additional PCAs: Mesoamerica, vintage 1, vintage 2 and early breeding lines (Additional file 5: Figure S3A and B). The SLL fresh group was comprised of the subgroups fresh 1, fresh 2 and vintage/fresh (Figure 2C, Additional file 5: Figure S3C and D). The latter subgroup was named vintage/fresh because it included accessions classified as vintage as well as contemporary breeding fresh market accessions. The SLL fresh 1 was composed of Florida and North Carolina accessions while SLL fresh 2 consisted of accessions from New York (Additional file 1: Table S1). The SLL processing 1 group was subdivided into three groups, 1–1, 1–2 and 1–3. The latter group was comprised of a subset of accessions from the Ohio breeding germplasm whereas the remainder of the Ohio germplasm was found in the SLL processing 1–2 subgroup. The processing 1–1 included accessions from Oregon. The group SLL processing 2 was clearly separated from the other processing groups. This group was entirely composed of New York breeding materials which represent a predominately California genetic background with *Phytophthora* resistance introgressed from North Carolina fresh-market accessions. Finally, the SLL1 group was located between SLL processing 1 and SLL fresh in the PCA (Figure 2C) and was comprised by a mixture of accessions such as the plum tomatoes Rio Grande and NC EBR-6.

To determine the consistency of the structure obtained by PCA analyses, we compared the distribution of genetic distances within the following hierarchy levels: species, genetic group, genetic subgroup and samples of the same uniquely named accession (Additional file 7: Figure S5). As expected, the species showed the highest distances whereas the groups and subgroups showed progressively lower genetic distance values. All pairwise genetic differentiation among subgroups assessed by F_st_ and D_st_ were significant (p-value < 0.05) (data not shown). The distance among repeated samples of the uniquely named accessions was very low indicating a high consistency among genotyping experiments.

### Comparison of the genetic and passport classifications

**Figure 4:**
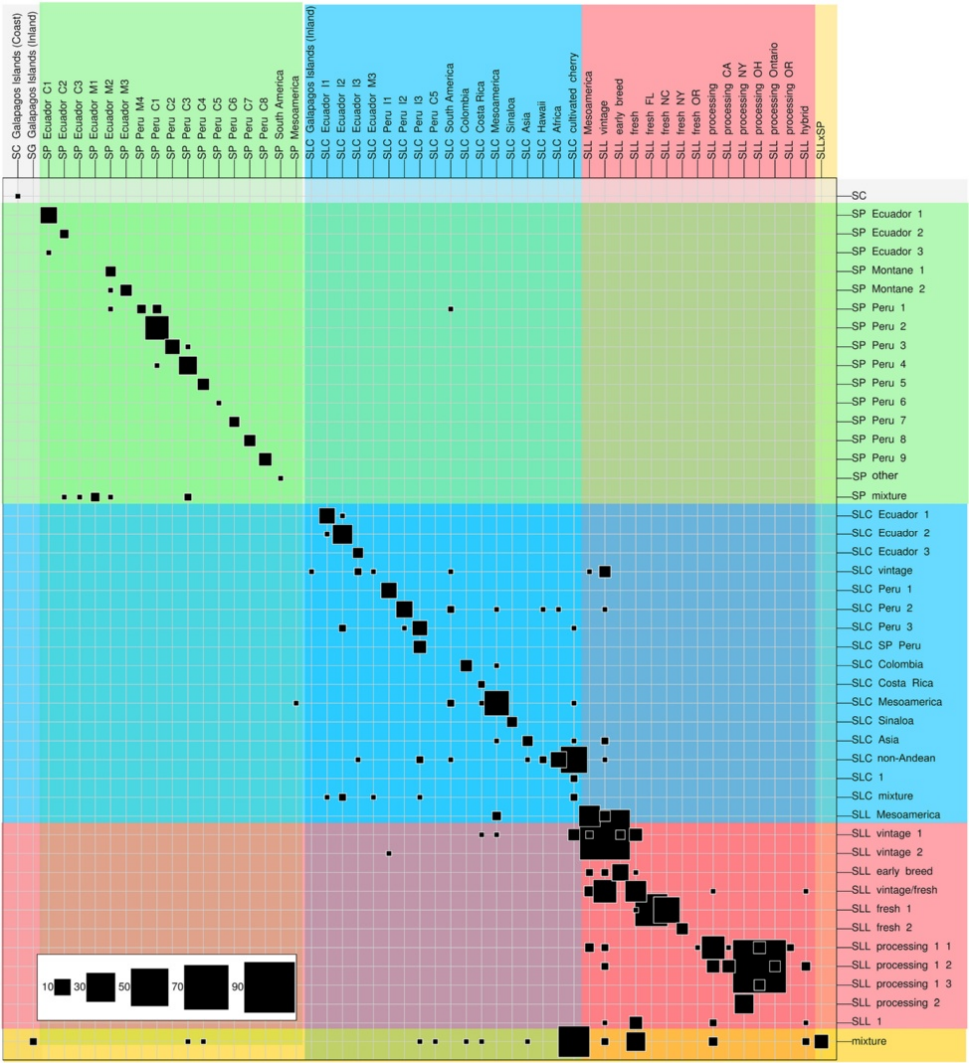
**Comparison between the passport-based classification (columns) and genetic-based classification (rows).** The genetic classifications correspond to the clusters shown in Additional file 1: Table S1 and, passport classification is based on information provided (see Material and Methods for further details). Size of the squares is proportional to the number of samples corresponding to each genetic and passport group and, background colors highlight different species and botanical varieties.

The genetic classification derived from the PCAs was compared with the passport-based classification and demonstrated overall good agreements (Figure 4 and Additional file 1: Table S1). Most disagreements were in SLC followed by SLL (Figure 4). One striking difference between the two classifications occurred for 102 SLC accessions that were located in the PCA between SLC and SLL and classified as mixture (Figure 1). These accessions included many of the commercial cherry tomatoes. These data imply that most cultivated cherry tomatoes are not true SLC.

One of the other notable exceptions to the correspondence between genetic and passport classification was the subgroup comprised of accessions that were listed as SLL vintage, but instead were genetically classified as a SLC group closely related to SLC Ecuador. This cluster was classified as SLC vintage and consisted of genetically diverse germplasm that included accessions collected mostly at South American markets.

### Population relationships

**Figure 5:**
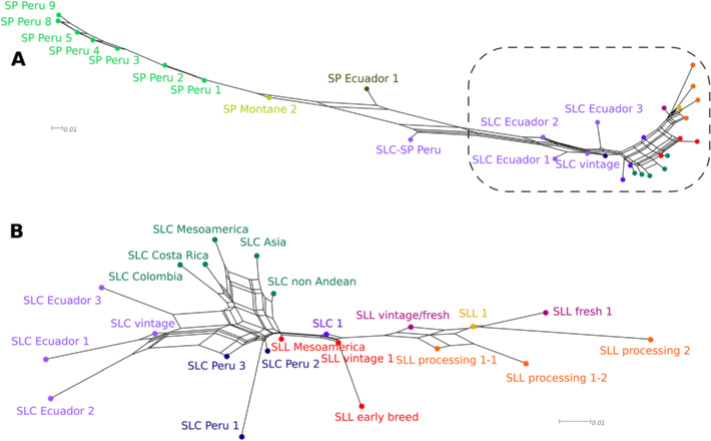
**Neighbor network for the genetic subgroups; Neighbor network based on genetic distances (D**_**est**_ **) for the genetic subgroups.** **(A)** Complete network and **(B)** close up of the region squared in panel **A**. Only subgroups with more than 5 individuals are represented. Different colors represent genetic groups.

To determine the relationship between all subgroups, we constructed a neighbor network and population phylogenetic tree reflecting subgroup relationships based on D_st_ distances (Figure 5A and Additional file 8: Figure S6). All SP subgroups clustered together presenting a latitudinal ordination from south to north in both the network and the phylogenetic tree. Little reticulation appeared in SP compared with SLC and SLL in the network, suggesting less gene flow between subgroups in SP. The closest to SLC in the network were the SP Montane 2 and SP Ecuador 1 subgroups. The group SLC-SP Peru was located at a genetic position between Ecuadorian SLC and SP and appeared to be the result of an admixture between these two species. Within SLC, groups that were found in close geographical proximity also tended to cluster together. The neighbor network showed two plausible paths for the evolution of SLC to SLL: 1) SLC Ecuador 3, SLC Colombia, SLC Costa Rica, SLC Mesoamerica; and 2) SLC Peru 1, SLC Peru 2, SLC Peru 3 and SLL Mesoamerica (Figure 5B). SLL groups also showed that SLL vintage and early breeding lines are genetically closely related to Mesoamerican SLL. The SLL fresh and SLL processing subgroups were more distant from the Mesoamerican and vintage SLL with evidence of reticulation. In general, the accordance between the proposed hierarchical genetic classification which is represented in the neighbor network and the population tree was high (Figure 5 and Additional file 8: Figure S6).

**Figure 6:**
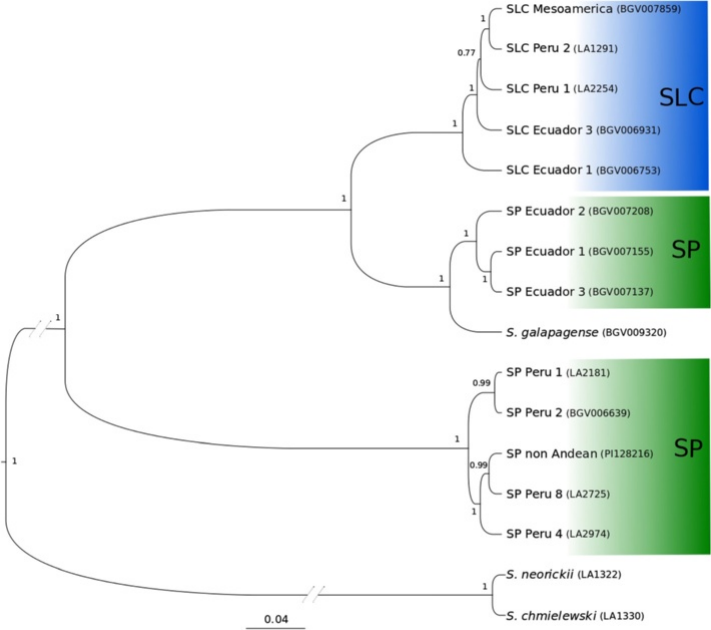
**Phylogenetic tree based on SNP data.** Phylogenetic tree based on SNP data computed with Bayesian based SNAPP algorithm. Posterior support of nodes is shown.

The accession-based phylogenetic tree that included *S. chmielewski*, *S. neoricki* and *S. galapagense* (Figure 6) showed that the Peruvian SP groups were basal for the red-fruited group, and Ecuadorian SP was phylogenetically the closest to SLC with SLC Ecuador 1 basal to the entire SLC. Interestingly the *S. galapagense* (SG) accession clustered very close to the Ecuadorian SP, a grouping which was also found in the PCA (Figure 1).

### Isolation by distance and climate

We noted that most clusters in SP and SLC corresponded to the location of where the accessions were collected. Therefore, we sought to evaluate the significance of this finding by calculating the correlation between genetic, climatic and geographic distances (Table 3). The highest correlation was found in SP indicating a strong positive correlation between genetic and climatic distances (*r* = 0.67, Mantel p-value = 0.01), as well as for genetic and geographic distances (*r* = 0.53, Mantel p-value = 0.01) (Additional file 9: Figure S7). Two sets of accessions were explored in SLC, one including the subgroups from Ecuador and Northern Peru and the other including SLC non-Andean. For the Ecuadorian and Northern Peruvian SLC, the relationship between genetic and climatic distances was lower (*r* = 0.29, Mantel p-value = 0.01) than in SP, whereas the genetic *versus* geographic was similar (*r* = 0.49, Mantel p-value = 0.01). When considering the SLC accessions together, a low correlation between genetic and climatic (*r* = 0.11, Mantel p-value = 0.09) as well as genetic and geographic distances (*r* = −0.19, Mantel p-value = 0.01) were observed.

**Table 3:** **Isolation by distance and climatic distance: correlation between climatic, geographic and genetic distances in SP, SLC Ecuador and Northern Peru and SLC non-Andean: number of accession (** ***n*** **), correlation coefficient (** ***r*** **) and p-value for Mantel test is shown**

	**Climatic vs. genetic**			**Geographic vs. genetic**		
	**n**	**r**	**p-value**	**n**	**r**	**p-value**
**SP**	96	0.67	0.01	106	0.53	0.01
**SLC Ecuador/N Peru**	65	0.29	0.01	79	0.49	0.01
**SLC non-Andean**	101	0.11	0.09	165	−0.19	0.01

### Diversity and heterozygosity

Expected heterozygosity (H_e_) and observed heterozygosity (H_o_) decreased in the succession from SP to SLC and SLL (Table 2 and Additional file 10: Figure S8). For SP, the SP Peru group retained the highest diversity followed by SP Montane and SP Ecuador. The Ecuadorian and Peruvian SLC (SLC Ecuador and SLC Peru) showed higher level of diversity (H_e_ = 0.19 and 0.18) compared to SP Ecuador and SP Montane. In contrast with the high diversity of the Ecuadorian and Northern Peruvian SLC, the other SLC subgroups exhibited low diversity, similar to that found in vintage SLL. For SLL a similarly low level of observed heterozygosity was typical for most subgroups. However, when combining the contemporary SLL subgroups (processing and fresh), slightly higher levels of diversity were found when compared to SLL vintage (H_e_ = 0.12 *vs.* 0.09), a situation that is likely due to the effect of introgression during breeding and differentiation into distinct market classes (Additional file 10: Figure S8).

**Figure 7:**
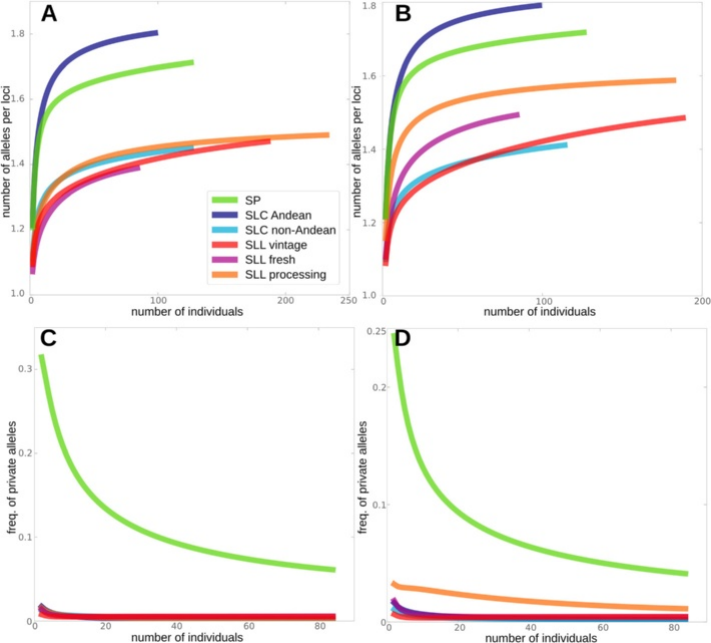
**Rarefaction analysis of the number of alleles per locus and frequency of private alleles.** Rarefaction analysis of the number of alleles per locus **(A, B)** and frequency of private alleles **(C, D)** for SP, SLC Andean (Ecuadorian and Northern Peruvian SLC), SLC non-Andean, SLL vintage, SLL fresh and SLL processing for two sets of markers. **A** and **C** show the results for a set of 2,312 markers spread at least 0.1 cM and **B** and **D** for 6343 SNPs (see text for details). Include which genetic subgroups are included in each category.

To avoid biases in the genetic diversity estimates due to the different number of individuals per group, a rarefaction analysis was carried out (Figure 7). To explore whether genetic diversity estimates might be inflated due to introgressed genomic segments from wild relatives present in contemporary SLL accessions, we conducted parallel analyses with two sets of markers. The first set included one marker every 0.1 cM (2,313 SNPs) (Figure 7A and C) and the second set included 6,343 SNPs, after removing monomorphic SNPs and SNPs with more than 10% of missing data (see Materials and Methods) (Figure 7B and D). When using the smaller marker set, the average number of alleles per locus of SP and the combined set of SLC Northern Peru and Ecuador was higher than in all other clusters (Figure 7A). When all markers were used, the SLL fresh and SLL processing showed an allele richness that was intermediate between Andean SLC and SP on the one hand, and non-Andean SLC and SLL vintage on the other (Figure 7B). When all SLL contemporary accessions were combined into one group, the analysis with the smaller marker set showed a slight increase in allelic richness compared to separate analyses of the SLL processing and SLL fresh genetic groups (Additional file 11: Figure S9A). Using all markers, the allelic richness of the combined contemporary accessions approached that of SP (Additional file 11: Figure S9B). These findings suggested that introgressions found in the contemporary accessions might lead to increased estimates of genetic diversity but also that differentiation into distinct market classes increased genetic divergence within SLL. Frequency of private alleles was also explored for the subset of markers (Figure 7C) and the whole dataset (Figure 7D). The highest proportion of private alleles was found in SP regardless the marker dataset used, whereas the number of private alleles was virtually the same for all other groups, except for the processing group when using the complete marker set. This finding might indicate the presence of introgressions from genetically diverse relatives in SLL processing.

LD was estimated between markers at different genetic distances from one another (Additional file 12: Figure S10). From highest to lowest degree of disequilibrium the groups were: fresh, processing, vintage, Andean SLC, non-Andean SLC and SP. These results suggest that LD affects estimates of allelic richness, especially when dealing with groups with different degrees of LD.

### Origin and migration of the derived tomato fruit shape and weight alleles

**Figure 8:**
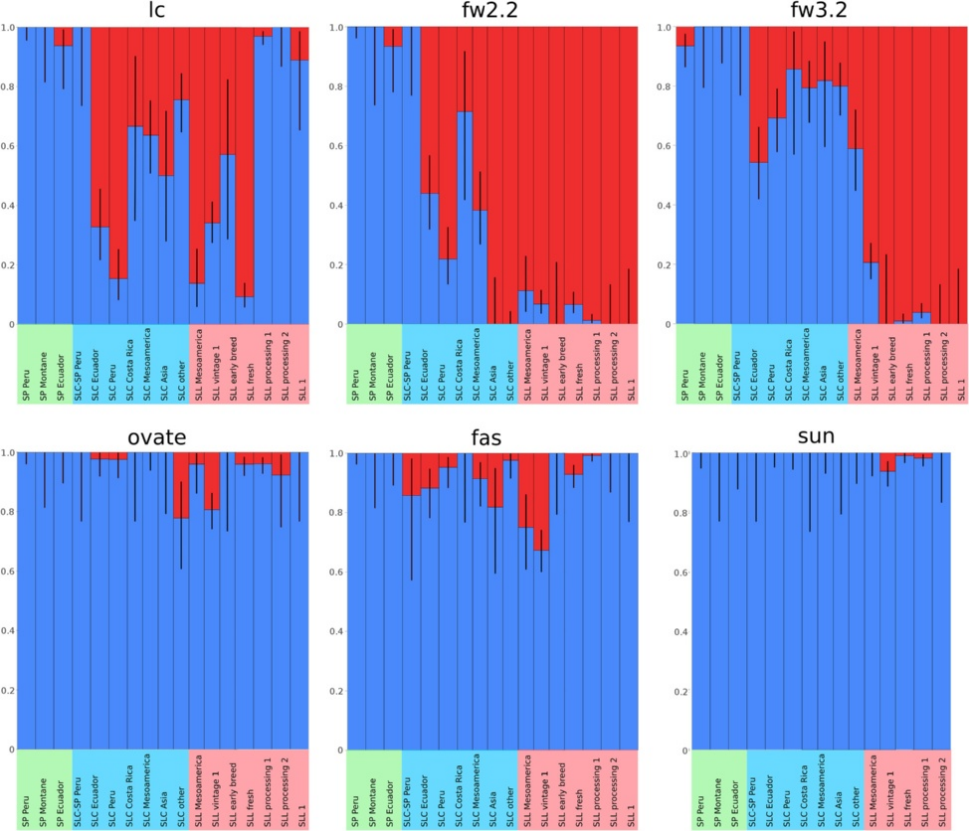
**Fruit weight and shape gene frequencies across genetic groups.** Ancestral allele in blue, derived allele in red. Black lines show binomial confidence intervals at 95%.Indicate which genetic subgroups are pooled in each category.

Several genes involved in the transition from small and round to large and variably shaped tomato have been cloned in recent years. In all cases, the nucleotide polymorphism that is associated with the change in fruit appearance is known. We wanted to investigate when and where the derived alleles of the six fruit shape and weight loci arose and how they migrated through populations in the evolution of tomato. For all fruit morphology loci, the derived allele was at very low frequency or not found in the SP accessions (Figure 8). The derived alleles for the *fw2.2* and *lc* loci were both found at very low frequency in SP Ecuador but at much higher, 55% or more, frequency in the Andean SLC groups (SLC Peru and SLC Ecuador). The *lc* mutation was also common in SLL vintage and SLL fresh accessions whereas the derived allele was not found in the SLL processing types. The derived allele of *fw2.2* was nearly fixed in all SLL groups. For *fw3.2*, the derived allele was found in SLC Ecuador and SLC Peru albeit at lower frequency compared to *lc* and *fw2.2*. Fixation of the derived allele did not occur in the SLL vintage but instead became nearly complete in the contemporary SLL accessions. The derived alleles of *fas* and *ovate* were most likely to have arisen in the Ecuadorian or Peruvian SLC accessions and were maintained at low frequency in the remaining SLC accessions. Of the SLL vintage, 20 and 30% carried the derived alleles of *ovate* and *fas*, respectively. In other SLL groups, the derived allele for *ovate* and *fas* were found at low frequency in this dataset. However, the derived allele of *ovate* is quite common among Italian vintage cultivars where 38 to 47% of the accessions carry the mutation [30]. *Sun* is present at low frequency in SLL vintage, fresh and processing whereas the allele has neither been detected in Ecuadorian and Peruvian SLC nor in the Mesoamerican accessions.

## Discussion

Key questions regarding the evolutionary history of cultivated tomato include where and when the crop was domesticated and the position of SLC in this evolutionary process. In this study, we interrogated a selection of 2,313 SNPs from the SolCAP array in nearly 1,000 unique accessions comprised of SLL, SLC and the red-fruited wild relative SP. By combining accessions with robust passport data we were able to test hypotheses about the origin of cultivated tomato. Our results support the two-step domestication hypothesis proposed by Blanca et al. [9], and are in line with recently published work about the origin of tomato [54]. As expected, genetic diversity was high in SP (Table 2), and genetic clusters were explained by geographic distances and climatic zones (Table 3 and Additional file 9: Figure S7). The higher number of SP accessions analyzed when compared with previous studies has allowed a more detailed definition of the SP populations, especially in Southern Peru, where sequential colonization could be proposed based on the PCA (Additional file 3: Figure S1) and the network analysis (Figure 5). SLC accessions from Ecuador and Peru also showed genetic structure that correlated with geography (Table 3 and Additional file 9: Figure S7). Genetic diversity was high in Ecuadorian and Peruvian SLC, but was reduced in SLC from Mesoamerica and elsewhere. Our results suggest that the major genetic bottleneck did not occur due to transport of SLL from Mesoamerica to Europe, but occurred earlier coinciding with the migration of SLC from Ecuador and Northern Peru to Mesoamerica (Table 2 and Additional file 9: Figure S7). In wild populations there is a strong correlation between geography, climate, and genetic distances (Table 3 and Additional file 9: Figure S7). These correlations do not occur in the non-Andean SLC and the SLL genetic subgroups, a situation that is common for plants associated with human activities, either cultivated or weedy, due to the movements of the seeds by the humans and to the artificial modifications or their environments [55,56].

### Phylogenetic relationships

The phylogenetic tree (Figure 6), neighbor network analyses (Figure 5) and the high number of private alleles (Figure 7) support the status of SP as the basal group of the red-fruited species of the *Lycopersicon* section. Our data also supports the view that the Northern Ecuadorian SP is the closest ancestor of SLC. Northern Ecuadorian SLC was likely to have originated from Ecuadorian SP, yet its high genetic diversity and its reticulate structure in the phylogenetic network suggests a complex history. The position of SG within SP contrasts with a recent study [57] in which SG was found to be closer to SLC than SP. However, firm conclusions about the position of Galapagos accessions will require further study, as both studies, Koenig’s *et al.* and the present one, are based on a few number of accessions and Koenig *et al.* lacked SP accessions from Ecuador.

The data suggest two possible scenarios for the origin of SLC. Ecuadorian SLC features twice the level of the genetic diversity as Ecuadorian SP (Table 1), thus it is not likely that SLC was simply derived from this SP subgroup, despite being very close phylogenetically. One hypothesis is that the subgroup named Peruvian SLC-SP represents the origin of SLC. This genetic subgroup is also close genetically to Ecuadorian SP (Figure 6). However the large geographic distance separating these subgroups challenges this scenario. It is possible that the Peruvian SLC-SP is the result of a secondary contact between SLC and SP. The second hypothesis is that ancestral populations of Northern Ecuadorian Coastal SP gave rise to SLC in Northern Ecuador across the Andes. Secondary gene flow between other SP populations, suggested by the reticulation of the phylogenetic network and the complex PCA structure, e.g. Montane SP from Southern Ecuador and Northern Peru may have enhanced diversity of the SLC. Alternatively, the sampling of Northern Ecuador SP may have been incomplete or an ancestral highly diverse population might have originated both Northern Ecuadorian SP and SLC. Ecologically, it is more plausible that SLC originated from Northern Ecuadorian SP. These Northern Ecuadorian SP accessions thrive in wet and forested areas of Coastal Ecuador, a climate closer to the wet environment on the Eastern side of the Andes where Ecuadorian SLC is found. In contrast, Peruvian SP is adapted to an arid climate. Climatic similarity of some SP and SLC populations may have facilitated gene flow due to animal or human movement, despite geographic differences. Previously, possible mechanisms for gene flow between SP and SLC have been proposed for this region [9,58].

The Mesoamerican SLL vintage subgroup appeared to be the most ancestral SLL according to the phylogenetic trees and the network. This SLL genetic subgroup was closely related to SLC Peru 2 in the phylogenetic network and tree. Thus, our data clearly support that SLC evolved into Mesoamerican SLL. According to the analyses with this dataset, all other SLL are monophyletic and all SLL groups originated from the SLL Mesoamerican accessions.

### Proposed origin and domestication based on derived alleles for fruit weight and shape

The most ancestral SLC is found in Ecuador and Northern Peru and it is characterized by a high genetic diversity and morphological variability [9]. It spans a wide range of domestication (from accessions collected in markets, and presumably cultivated at production scale, to weeds) and use (from human consumption to animal feed), which suggests a certain degree of selection for SLC. This finding is supported by the fact that the derived alleles of *lc, fw3.2* and *fw2.2* are already prevalent in the ancestral SLC accessions from Northern Peru and across Ecuador (Figure 8). The derived allele of *lc* and *fw2.2* may have originated in the Ecuadorian SP and could represent the earliest known mutations to arise. However, this interpretation needs to be viewed with caution as only two SP accessions carrying a single derived allele of each locus were identified (Additional file 1: Table S1). Interestingly, the SLC vintage group that clusters closely with Ecuadorian SLC included accessions that were collected from markets and feature fruits that are large, ribbed and multi-loculed (Figure 3). The strongest selection may have taken place in this subgroup as all accessions carried the derived alleles for *lc*, *fw2.2* and *fw3.2*, and half of them carried the derived allele of *fas* (Figure 3 and Additional file 1: Table S1). None of the other SLC subgroups were fixed for as many fruit weight and shape alleles as the SLC vintage category. Thus it appears that SLC was being cultivated and that selections for larger fruit were taking place (Figure 3 and Additional file 1: Table S1). SLC Mesoamerica carried derived and ancestral alleles for most of the fruit shape and weight loci, while SLC Asia and SLC Other were completely fixed for the derived allele of *fw2.2* suggestive of selection for the SLC germplasm grown outside the Americas.

SLL arose in Mesoamerica as there is no evidence of the existence of ancestral SLL in South America. All SLL accessions sampled from South America were found to carry introgressions from wild relatives suggesting that they were derived from breeding efforts taking place in the last 100 years. Therefore, to complete the domestication of SLL, SLC would have had to migrate to Mesoamerica possibly as a semi-domesticated type. According to the network analysis, PCA results, and previous knowledge of species history two SLC migrations could be suggested. SLC could have migrated from Southern Ecuador to Colombia and Costa Rica arriving in Mesoamerica in a stepwise process (Figure 2D). However, a second possibility is also suggested by our results, SLC could have reached Mesoamerica from Northern Peru in one step. Fruit weight and shape allele distribution did not support one route of migration over the other. In any case, results from the gene diversity analysis suggest that the migration from the Ecuador or Northern Peruvian region to Mesoamerica led to a strong bottleneck which eventually resulted in reduced variation in Mesoamerican SLL, as described by Blanca et al. [9]. The second phase of tomato domestication in Mesoamerica is suggested by the increase in derived allele frequency for *fw3.2*. Allele frequencies for fruit weight loci suggest that selection for *fw2.2* and *lc* were important for the origin of SLC while *fw3.2* was important for the origin of SLL. Our results agree with a recent study [54] based on 360 tomato genomes. They also find evidence for a two-step domestication, and identify new QTLs implicated in both steps of domestication and breeding.

The American origin of the first European tomato is confirmed by the genetic relationship between the Mesoamerican and vintage SLL subgroups (Figure 6). It is remarkable that the vintage SLL appears to have been derived exclusively from Mesoamerican germplasm. Although large fruited vintage SLC were found in South America, they did not appear to contribute to the germplasm that migrated to Europe and the rest of the world. It is not possible with the current data to know why the Ecuadorian and Peruvian SLC did not contribute to the Spanish vintage gene pool brought to Europe, despite being those regions also under the control of the spaniards, but we could propose that climatic similarity between Mexico and Spain could have played a role.

### Contemporary tomato diversity

Since the introduction of the modern breeding in the 20th century, the pace of genetic change in SLL has accelerated. New germplasm has been created that, according to the PCA, network and population tree, differ substantially from the vintage accessions. These results are consistent with previous findings [19-22,31]. The contemporary tomatoes can be differentiated into four broad groups: fresh, processing 1, processing 2 and SLL1. This broad differentiation among the contemporary groups reflects independent breeding efforts and selection histories between the fresh and processing accessions. The further subdivision of the contemporary groups can be explained by geographic origin or founder effects in regional breeding programs. Similar results were previously reported by Sim et al. [21,31]. These subgroups differentiate accessions coming from the main public-sector breeding programs in North America. For processing they were historically carried out in California, the Midwest of the United States, the East Coast of the United States and Ontario, Canada. These programs commonly interchanged breeding materials, thus it is to be expected that the genetic groups mix those origins, albeit in different proportions [21]. The neighbor network reticulation found in these subgroups is compatible with this history (Figure 5).

Contemporary tomatoes are the result of introgressing genes from wild species into SLL starting before 1920 [59]. The PCA and rarefaction analyses (Figure 7) provided insight into the effect of these breeding practices on diversification and structure of the cultivated species, showing the existence the large introgressions as has also been described recently [54]. When we consider all contemporary accessions as a single gene pool (e. g. both fresh and processing markets), they represent a slightly more diverse population than the vintage tomatoes (H_e_ 0.12 *vs* 0.09, respectively) (Additional file 11: Figure S9). However, the differential overestimation of the gene diversity estimates within SP, SLC and SLL depending on the number of markers used, should be taken into account in future studies, especially now that thousands of markers are routinely being used in genotyping by sequencing (GBS) and whole genome sequencing experiments. Contemporary breeding seems to have moderately increased the variation and diversity in cultivated tomato, although it remains low when compared with the most ancestral SLC subgroups. These Ecuadorian and Peruvian accessions may represent a pool of unexplored variation for future improvement.

The distribution of the fruit weight and shape alleles is skewed in processing and in fresh market classes of SLL, suggesting high selection pressures of shape loci for each market class. In contrast, the distribution of these alleles is more varied in the vintage group which is why this class is characteristically variable in shape and weight [30] while contemporary material is quite uniform [60]. The observation of more phenotypic diversity for shape and weight, and more allelic diversity for these six loci in the vintage class appears to contrast with the observation of more genetic diversity in the contemporary germplasm. Contemporary market classes are bred for uniformity of shape and weight within a class, as reflected by the allele distribution for *lc*, *fas*, *fw2.2* and *fw3.2*. At the same time there are numerous resistances that have been bred into germplasm, with any given accession having multiple introgressed alleles that are missing from the vintage class, hence increased genetic diversity instead of phenotypic diversity in the contemporary class.

## Conclusions

This work represents an effort to show a comprehensive view of genomic variation in tomato and closely related species. We have analyzed and classified 1,008 tomato accessions, including the complete set of its closest wild relative, *S. pimpinellifolium.* The data are an excellent resource for evolutionary biologists and plant breeders. Our analysis support a two-step domestication as proposed by Blanca et al. [9]; a first domestication in South America and a second step in Mesoamerica,. The distribution of fruit weight and shape alleles also supports these two steps and shows that domestication of SLC occurred in the Andean region of Ecuador and Northern Peru. The definition and clarification of the biological status of SLC is also an important result of this work.

### Availability of supporting data

All supporting data are included as supplementary files.

## Additional files

Additional file 1: Table S1. Sample list, passport data and genotyping data for fruit shape and weight (SUN, LC, OVATE, FAS, FW2.2 and FW3.2). A, wild allele; B, derived allele and A/B, heterozygous. Additional file 2: Table S2. Genotyping data. Additional file 3: Figure S1. Principal component analysis for SP. Same legend as Figure 2. Additional file 4: Figure S2. Principal component analysis for SLC. Same legend as Figure 2. Additional file 5: Figure S3. Principal component analysis for SLL. Same legend as Figure 2. Additional file 6: Figure S4. Geographical distribution of genetic sub-groups in (A) the Andean Region, (B) Mesoamerica and of (C) species all around the world. Same legend as Figure 2. Additional file 7: Figure S5. Pairwise genetic differentiation between accessions and within genetic groups; Violin plot showing the distribution of pairwise genetic differentiation between accessions and within each genetic groups at different hierarchy levels of the genetic classification. Pairwise distances among different samples of the same accession are shown. Additional file 8: Figure S6. Neighbor-joining tree based on the population distances measured as D_est_ among genetic subgroups. Bootstrap values based on 1,000 trees are shown. Branches with a bootstrap support lower than 70 have been collapsed. Additional file 9: Figure S7. Correlation among genetic and climatic distance (A, B, C) and genetic and geographic distance (D, E, F) for SP accessions, SLC accessions from the Andean region and all SLC accessions. Solid lines show the result of the linear regression model. Different colors represent the density of comparisons. Additional file 10: Figure S8. Observed and expected heterozygosity (H_o_, H_e_) for the species (upper) and groups of the genetic-based classification (lower). Additional file 11: Figure S9. Rarefaction analysis of the number of alleles per locus and frequency of private alleles. Rarefaction analysis of the number of alleles per locus (A, B) and frequency of private alleles (C, D) for SP, SLC Andean (Ecuadorian and Northern Peruvian SLC), SLC non-Andean, SLL vintage and SLL contemporary (SLL fresh and SLL processing) for two sets of markers. A and C show the results for a set of 2,312 markers spread at least 0.1 cM and B and D for 6343 SNPs (see text for details). Include which genetic subgroups are included in each category. Additional file 12: Figure S10. Linkage disequilibrium (LD) measured as *r* ^*2*^ for SP, Andean and non Andean SLC and vintage, fresh and processing SLL against genetic distance between SNP markers within each chromosome. Curves represent the resulting fits to a LOESS model.
