# Supplementary figures and images for: Genomic variation in tomato, from wild ancestors to contemporary breeding accessions
Source: BMC Genomics. 2015 Apr 1;16(1):257. doi: 10.1186/s12864-015-1444-1 (PMC4404671; doi:10.1186/s12864-015-1444-1)

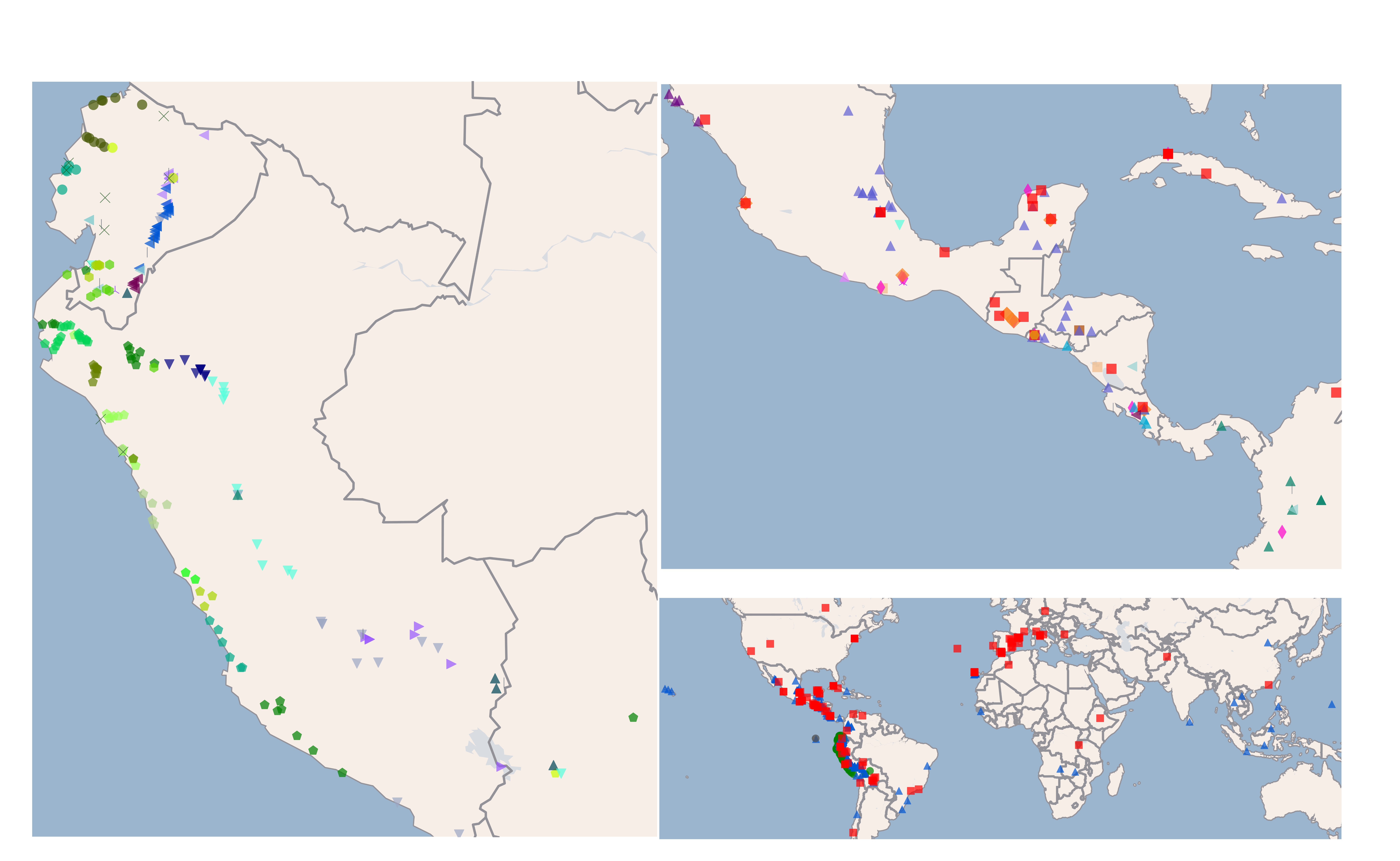

Supplement: Additional file 6: Figure S4. — Geographical distribution of genetic sub-groups in (A) the Andean Region, (B) Mesoamerica and of (C) species all around the world. Same legend as Figure 2. [file 12864_2015_1444_MOESM6_ESM.jpeg]

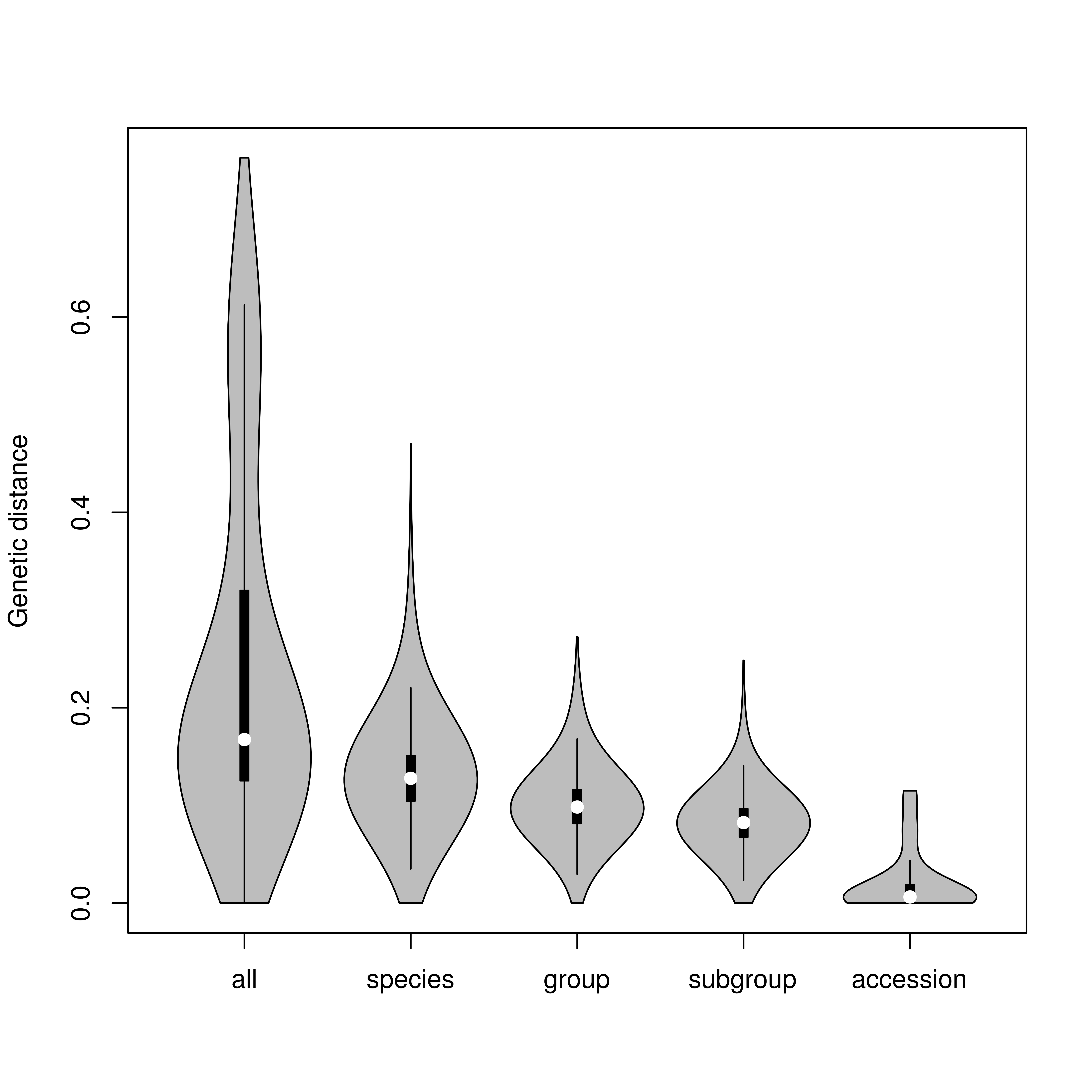

Supplement: Additional file 7: Figure S5. — Pairwise genetic differentiation between accessions and within genetic groups; Violin plot showing the distribution of pairwise genetic differentiation between accessions and within each genetic groups at different hierarchy levels of the genetic classification. Pairwise distances among different samples of the same accession are shown. [file 12864_2015_1444_MOESM7_ESM.jpeg]

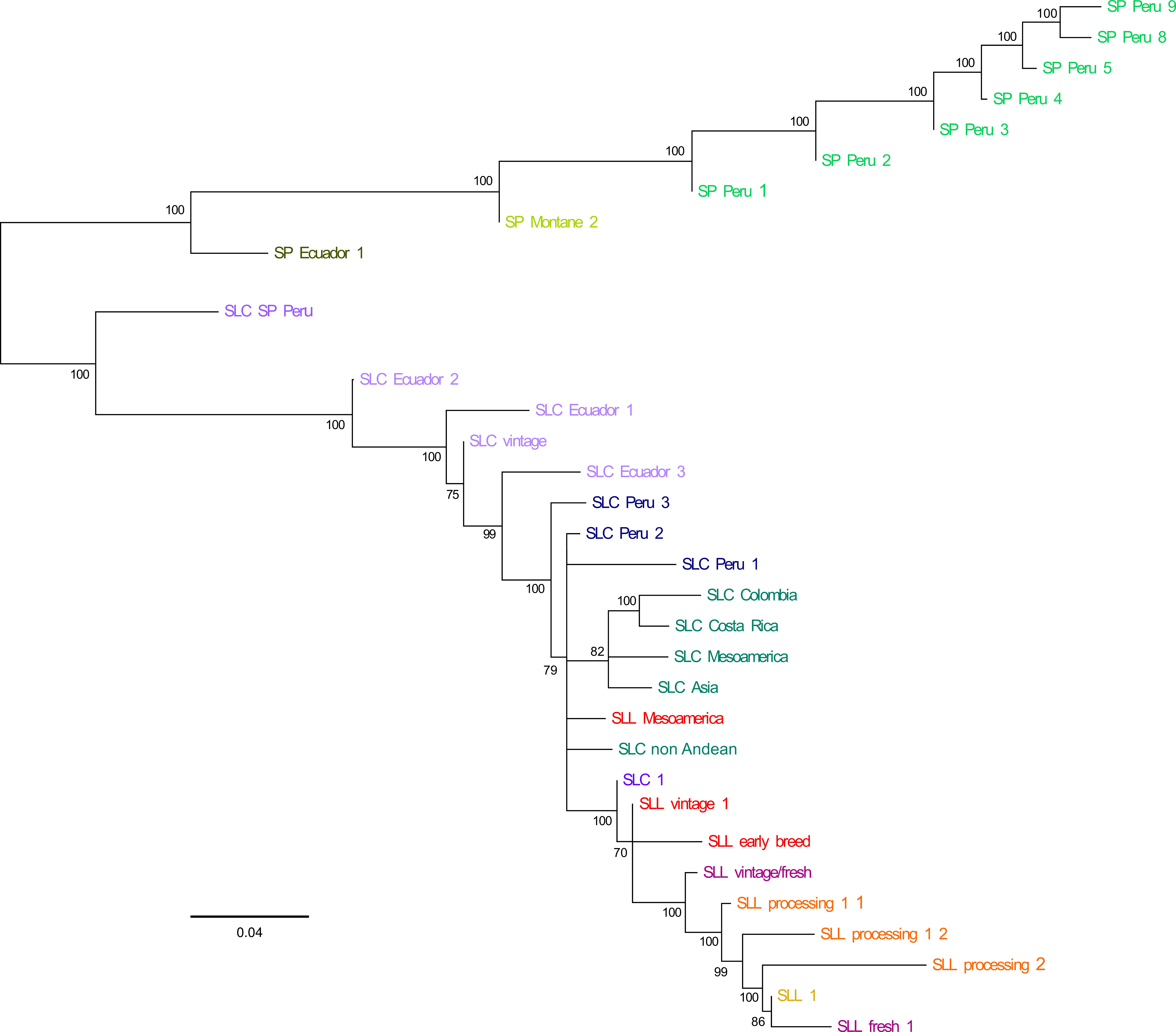

Supplement: Additional file 8: Figure S6. — Neighbor-joining tree based on the population distances measured as Dest among genetic subgroups. Bootstrap values based on 1,000 trees are shown. Branches with a bootstrap support lower than 70 have been collapsed. [file 12864_2015_1444_MOESM8_ESM.jpeg]

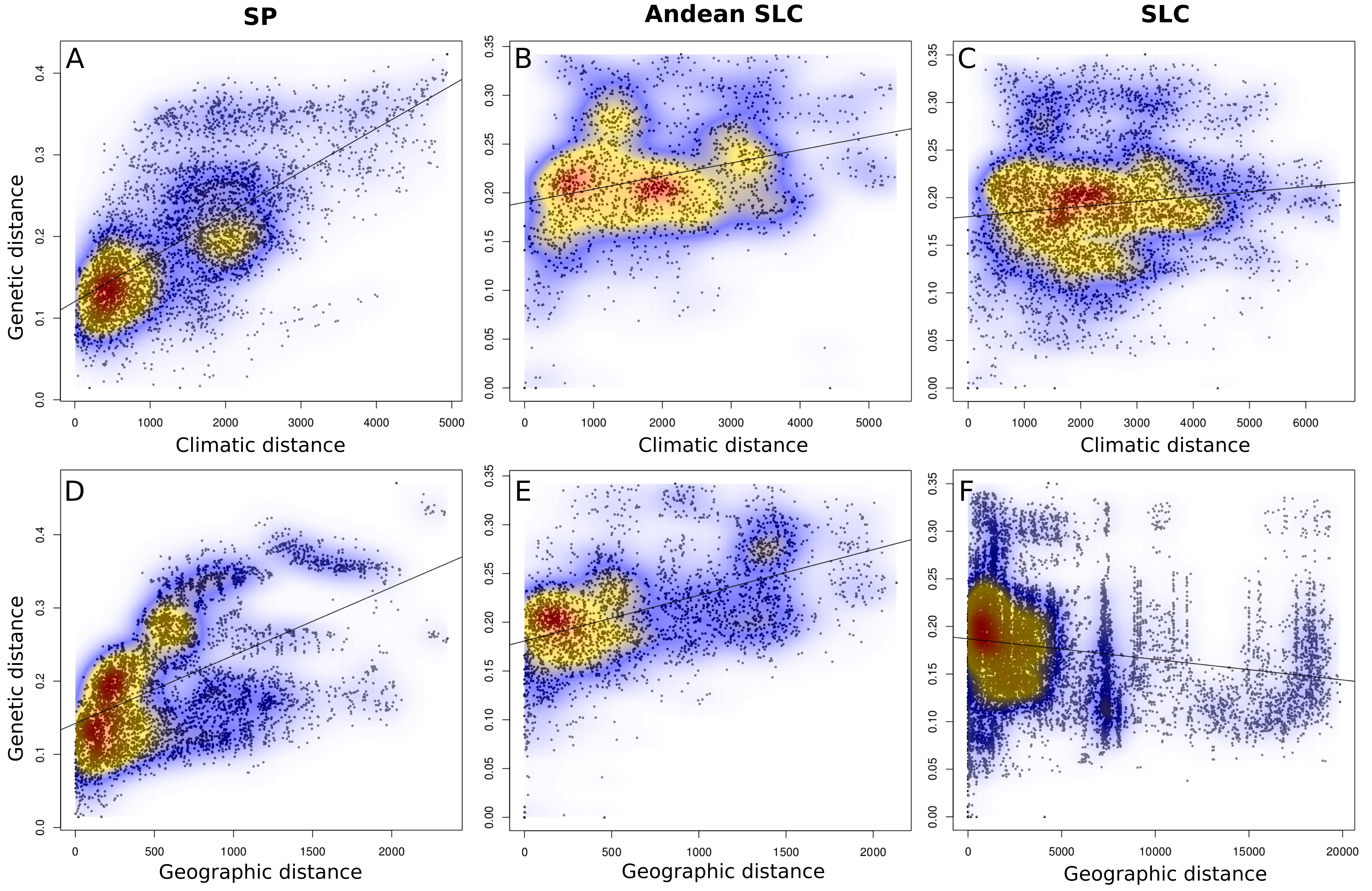

Supplement: Additional file 9: Figure S7. — Correlation among genetic and climatic distance (A, B, C) and genetic and geographic distance (D, E, F) for SP accessions, SLC accessions from the Andean region and all SLC accessions. Solid lines show the result of the linear regression model. Different colors represent the density of comparisons. [file 12864_2015_1444_MOESM9_ESM.jpeg]

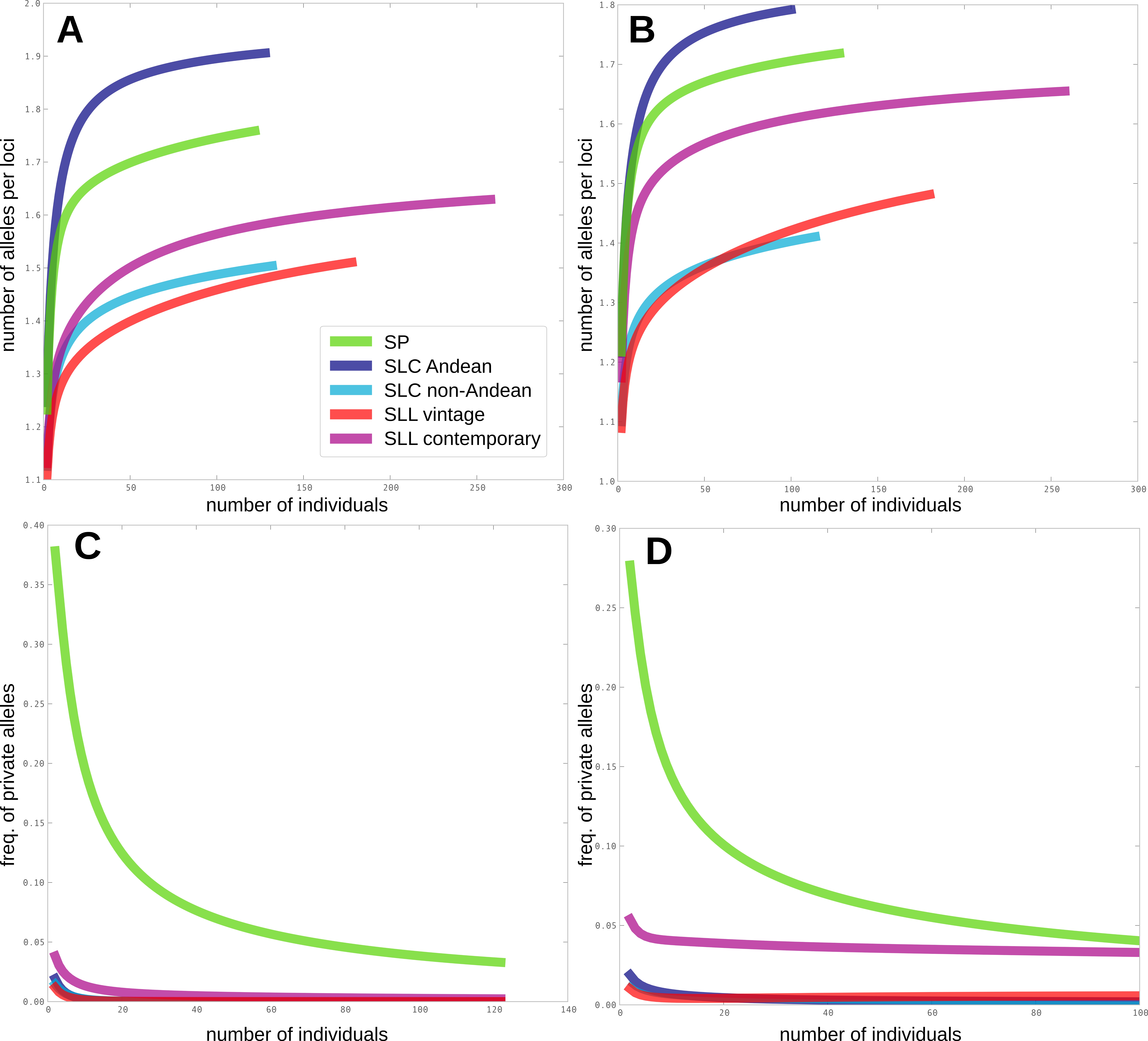

Supplement: Additional file 11: Figure S9. — Rarefaction analysis of the number of alleles per locus and frequency of private alleles. Rarefaction analysis of the number of alleles per locus (A, B) and frequency of private alleles (C, D) for SP, SLC Andean (Ecuadorian and Northern Peruvian SLC), SLC non-Andean, SLL vintage and SLL contemporary (SLL fresh and SLL processing) for two sets of markers. A and C show the results for a set of 2,312 markers spread at least 0.1 cM and B and D for 6343 SNPs (see text for details). Include which genetic subgroups are included in each category. [file 12864_2015_1444_MOESM11_ESM.jpeg]

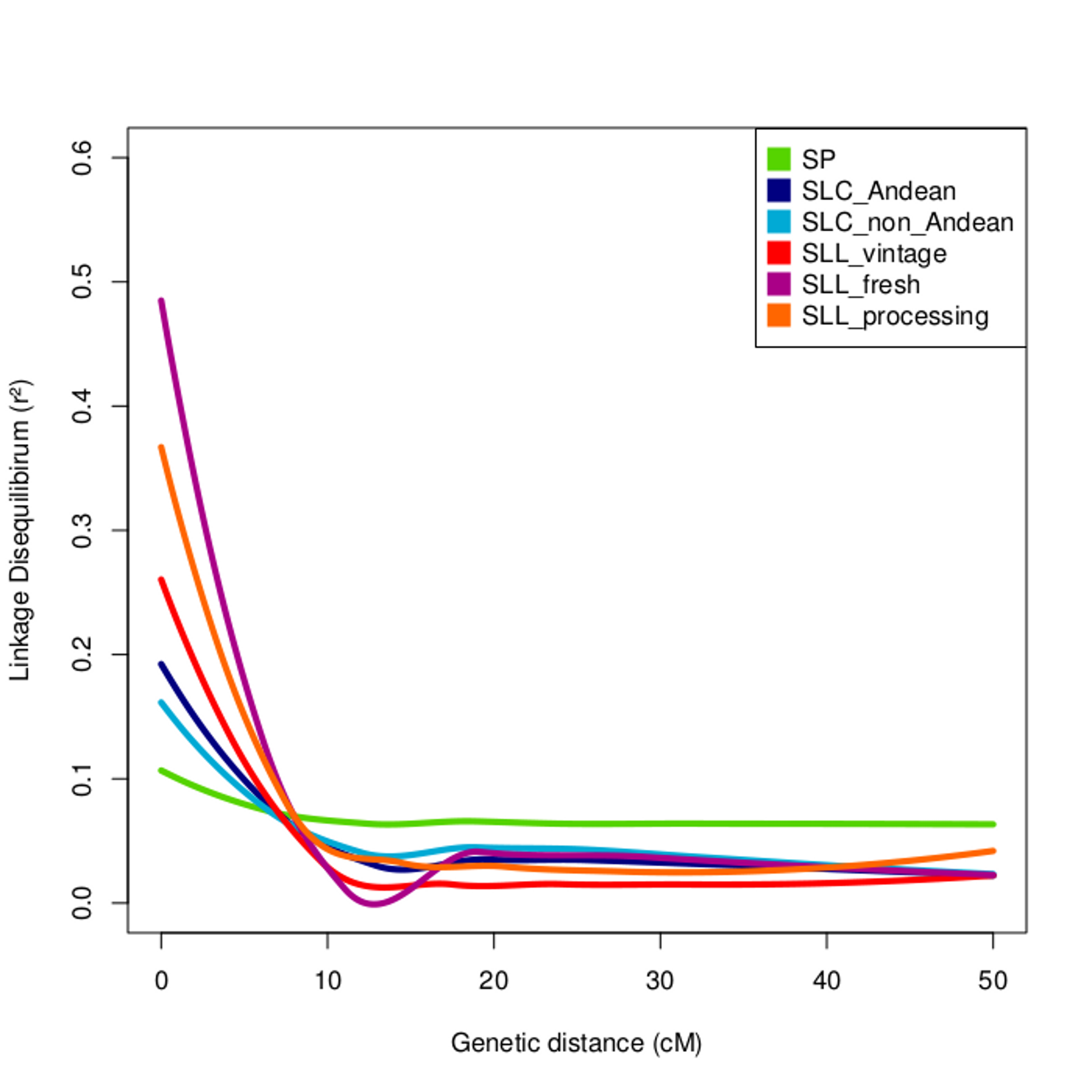

Supplement: Additional file 12: Figure S10. — Linkage disequilibrium (LD) measured as r 2 for SP, Andean and non Andean SLC and vintage, fresh and processing SLL against genetic distance between SNP markers within each chromosome. Curves represent the resulting fits to a LOESS model. [file 12864_2015_1444_MOESM12_ESM.jpeg]
